# User Experiences With Digital Future-Self Interventions in the Contexts of Smoking and Physical Inactivity: Mixed Methods Multistudy Exploration

**DOI:** 10.2196/63893

**Published:** 2025-06-20

**Authors:** Kristell M Penfornis, Nele Albers, Willem-Paul Brinkman, Mark A Neerincx, Andrea WM Evers, Winifred A Gebhardt, Eline Meijer

**Affiliations:** 1 Unit Health-, Medical and Neuropsychology Institute of Psychology Leiden University Leiden The Netherlands; 2 Department of Intelligent Systems Delft University of Technology Delft The Netherlands; 3 Department of Perceptual and Cognitive Systems Nederlandse Organisatie voor Toegepast Natuurwetenschappelijk Onderzoek Soesterberg The Netherlands; 4 Department of Public Health and Primary Care Leiden University Medical Center Leiden The Netherlands

**Keywords:** smoking, physical activity, future-self intervention, user experiences, multistudy, mixed methods

## Abstract

**Background:**

Smoking and physical inactivity compromise health, especially in combination. Interventions to promote smoking cessation and increased physical activity (PA) often lack impact, especially in the long term. Digital future-self interventions (FSIs), which prompt individuals to imagine who they do and do not want to become (ie, their desired and undesired future selves), show promise in encouraging sustainable changes in both behaviors. However, knowledge of user experiences with digital FSIs is limited. A deeper understanding of these experiences could help optimize FSIs, enhancing their efficacy in supporting smoking cessation and increased PA sustainably.

**Objective:**

This study examined behavioral, cognitive, and affective experiences with digital FSIs focused on smoking, PA, or both. Potential differences in user experiences based on behavior (smoking vs PA), polarity (desired vs undesired future self), and modality (verbal vs visual description of future selves) were explored.

**Methods:**

Secondary analyses of quantitative and qualitative survey data from 3 studies using digital FSIs as a means to encourage smoking cessation or increase PA were conducted. In study 1, participants (N=144) thought about how it would be to complete the FSI. In studies 2 (N=447) and 3 (N=87), they completed an FSI. Each study highlighted different aspects of user experiences with FSIs, namely, behavioral (eg, time spent), cognitive (eg, mental effort exerted), or affective (eg, emotions) experiences. Quantitative and qualitative findings were integrated for a comprehensive interpretation.

**Results:**

Regarding behavioral experiences, participants completed future-self tasks promptly (mean 6.64, SD 8.30 minutes), spent less time completing the desired- versus undesired-future-self (*P*<.001; η_p_^2^=0.227) and verbal versus visual (*P*=.03; η_p_^2^=0.060; quantitative) tasks, and integrated the tasks into their lives (qualitative). Despite tasks being preparatory and not actively encouraging behavior change, multiple participants reported implementing changes in their smoking or PA (qualitative). Regarding cognitive experiences, moderate effort (mean 5.85/10, SD 2.56) was exerted on the tasks regardless of behavior (*P*=.69; η_p_^2^=0.002), modality (*P*=.45; η_p_^2^=0.004), or polarity (*P*=.69; η_p_^2^=0.002; quantitative). Experiences of task difficulty were inconsistent across studies, individuals, and tasks, although mental visualization and describing one’s future self using images were consistently reported as challenging (quantitative and qualitative). Future-self tasks were reported to prompt cognitive processes such as contemplating consequences of smoking and PA behavior (qualitative). Regarding affective experiences, desired- and undesired-future-self tasks elicited different emotions (*P*<.001; η_p_^2^=0.630; quantitative). Desired-future-self tasks were perceived as enjoyable and happiness inducing, whereas undesired-future-self tasks were perceived as confronting and unpleasant, evoking feelings of sadness, fear, and anger (quantitative and qualitative).

**Conclusions:**

Digital FSIs appeared to be a time-efficient, feasible, and acceptable way of strengthening identities as a means to encourage smoking cessation and PA. Findings support continued implementation of digital FSIs, although further research is required to optimize their operationalization. Avenues in that regard are proposed and discussed.

## Introduction

### Background

Smoking is the leading behavioral risk factor for disease and premature death worldwide, and physical inactivity is the tenth [1]. Annually, tobacco is responsible for approximately 8 million deaths worldwide, and physical inactivity is responsible for approximately 1 million [2,3]. Combined, smoking and physical inactivity more than double the likelihood of premature death [4] and decrease disease-free life years by 6 years [5]. Consequently, interventions targeting both smoking cessation and physical activity (PA) have great potential to reduce worldwide mortality and morbidity, with multi-behavior interventions potentially enhancing effectiveness through synergistic effects [6,7]. Digital platforms such as eHealth and mobile health further enable scalable, accessible support [8,9].

Quitting smoking and increasing PA immediately mitigate health risks even among older individuals and those with long-standing histories of smoking or sedentary behavior [10,11]. However, quitting smoking and increasing PA are often challenging. To illustrate, long-term smoking abstinence may require 20 to 30 attempts [12]. While numerous smoking cessation and PA promotion interventions exist, their effects tend to be small to moderate and are rarely sustained beyond a year [13-15]. This leaves individuals vulnerable to relapses and renewed risks of negative health outcomes.

Identity-focused health behavior change interventions, which aim to connect healthy behavior to central components of who one is (ie, one’s self-identity), present a promising avenue to enhance the effectiveness of smoking cessation and PA promotion programs. Such interventions are rooted in identity theories that propose that, as people prefer to act in line with their self-identity [16,17], one is more likely to abstain from smoking or engage in regular PA when such healthy behavior is integrated into one’s self-identity. Empirical research supports this—individuals identifying with smoking show weaker intentions to quit [18,19] and are less likely to attempt cessation [20-22], whereas those identifying as quitters or nonsmokers are more likely to attempt [20,22] and achieve cessation [18]. Similarly, identifying with PA is linked to more intensive and frequent PA [23-26].

Among identity-focused interventions, future-self interventions (FSIs) appear particularly promising. FSIs are rooted in possible self–related theories, one branch of identity theory, which posit that possible future identities and not only current identity influence current behavior [27,28]. A clear vision of one’s desired or undesired future self can motivate self-regulatory processes to become the desired future self [28,29]. This is why FSIs generally prompt people to imagine who they want to become (ie, desired future self) or who they want to avoid becoming (ie, undesired future self). Research recommends envisioning both desired and undesired future selves for greater effect on identity and, subsequently, behavior [29,30]. Common tasks include writing about the envisioned future selves (ie, verbal future-self tasks), and searching for images that describe them (ie, visual future-self tasks) is also common [20,31]. Such tasks help create a more vivid image of the future self, ultimately increasing the likelihood of behaving in ways confirming or disconfirming that possible future identity [31]. FSIs are increasingly being delivered digitally [20,32].

Despite a solid theoretical foundation, experimental studies using FSIs in the context of smoking and PA have shown mixed results. While FSIs have repeatedly promoted PA [31,33-35] and impacted smoking cessation–related outcomes (eg, lower craving intensity, greater quit intention, and smoking reduction and abstinence [36-38]), some studies have found no effect on smoking [20,32] or PA [39]. The effectiveness of digital health behavior change interventions appears to hinge on user engagement [40]. Understanding user experiences is essential to determine the ideal type and level of engagement [41]. However, beyond one study reporting FSIs as useful to reduce smoking [37] and 2 reporting that future-self imagery can influence affect [42,43], little is known about user experiences with FSIs. Therefore, understanding user experiences is crucial for optimizing FSIs to facilitate smoking cessation and PA promotion.

### Objectives

Examination of user experiences is most complete when considering behavioral (eg, use and time spent), cognitive (eg, [mental] effort, interest, and attention), and affective (eg, emotional response) experiences [44-47]. Relying on a single study risks offering a narrow or skewed viewpoint influenced by the specific FSI examined. Therefore, this multistudy paper triangulates behavioral, cognitive, and affective user experiences across several digital FSIs aimed at facilitating smoking cessation or PA promotion to offer a more comprehensive overview.

The 3 digital FSIs in this study asked people to envision smoking- or PA-related desired and undesired future selves and complete verbal and/or visual tasks about these self-views. Given that smoking is a health-compromising behavior that is preferably quit and PA is a health-promoting behavior that is preferably increased, user experiences with FSIs may differ by behavior. Therefore, we explored potential differences in user experiences by behavior (smoking vs PA). As examining desired and undesired future selves jointly may mask varying experiences with one versus the other, we also explored differences in user experiences by task polarity (desired- vs undesired-future-self task). Third, because individuals may experience verbal and visual tasks differently, we explored whether user experiences vary by modality (visual vs verbal future-self task). Finally, to gain full insight into user experiences with FSIs, we explored interactions among behavior, polarity, and/or modality and also explored free-text responses.

## Methods

### Overview

This multistudy report is an observational, exploratory analysis of secondary quantitative and qualitative survey data from 3 empirical studies that used distinct digital FSIs. Combining quantitative and qualitative methods from multiple studies enabled data triangulation across contexts while providing deeper, more nuanced insights into individuals’ experiences [47-49]. The multistudy approach enhanced comprehensive understanding of experiences with FSIs and reduced the likelihood of chance effects. This study followed the Mixed Methods Article Reporting Standards. The 3 studies are, from this point on, referred to as study 1, study 2, and study 3. Personal characteristics of the participants included in the 3 studies are presented in Table 1.

The characteristics of the 3 original studies and outcomes used in this multistudy report to explore user experiences are presented in Table 2. Each study provides complementary insights into user experiences compared to the previous ones. Study 1 primarily explored cognitive experiences with a smoking- and PA-related desired- and undesired-FSI. Study 2 primarily explored cognitive and behavioral experiences with a smoking- and PA-related desired- and undesired-FSI and provided some insights into affective experiences as well. Study 3 explored behavioral, cognitive, and affective experiences with a smoking-related desired- and undesired-FSI. Collectively, the 3 studies offer a comprehensive understanding of experiences with FSIs.

**Table 1:** Personal characteristics of the participants included in studies 1, 2, and 3.

Characteristic			Study 1 (N=144)		Study 2 (N=447)		Study 3 (N=87)
Age (y), mean (SD; range)			37.55 (11.76; 20-69)		36.25 (11.44; 19-71)		37.84 (19.22; 18-82)
**Gender^a^** **, n (%)**							
	Men	72 (50)		211 (47.2)		27 (31)	
	Women	72 (50)		222 (49.7)		60 (69)	
	Nonbinary or other	0 (0)		14 (3.1)		0 (0)	
**Socioeconomic position^b^** **, n (%)**							
	Do not know	—^c^		3 (0.7)		—	
	Lower	1 (0.7)		2 (0.4)		13 (14.9)	
	Middle	32 (22.2)		126 (28.2)		45 (51.7)	
	Higher	111 (77.1)		316 (70.7)		29 (33.3)	
**Cigarette or e-cigarette consumption^d^** **, n (%)**							
	Once per day	5 (3.5)		25 (5.6)		1 (1.1)	
	2-5 times per day	97 (67.4)		73 (16.3)		28 (32.2)	
	6-10 times per day	48 (33.3)		93 (20.8)		21 (24.1)	
	11-19 times per day	45 (31.3)		104 (23.3)		13 (14.9)	
	≥20 times per day	25 (17.4)		152 (34)		24 (27.6)	
**Weekly exercise^e^** **, n (%)**							
	Never to little	30 (20.8)		124 (27.7)		—	
	Sometimes	80 (55.6)		193 (43.2)		—	
	Often	34 (23.6)		129 (28.9)		—	

^a^For studies 1 and 2, the “men” and “women” categories include transgender individuals.

^b^Lower: no formal education, primary education, and high school or equivalent; middle: tertiary education; higher: technical or community college or undergraduate, graduate, or doctoral degree as per the International Standard Classification of Education [50].

^c^This answer option was not provided in the study.

^d^For study 2, the numbers reflect smoking and vaping together.

^e^Never to little: 0 to 60 minutes per week; sometimes: 60 to 150 minutes per week; often: >150 minutes per week.

**Table 2:** Characteristics of the 3 original studies and outcome variables used to explore behavioral, cognitive, or affective user experiences with the corresponding future-self interventions.

		Study 1: cognitive experiences with a future-self intervention	Study 2: behavioral and cognitive experiences with a future-self intervention	Study 3: behavioral, cognitive, and affective experiences with a future-self intervention
**Design, measurements, and future-self intervention**				
	Study design	Online observational cross-sectional study	Online longitudinal observational study	Online longitudinal experimental study
	Data collection mode	Online survey	Online survey	Online survey
	Type of data	Quantitative and qualitative	Quantitative and qualitative	Quantitative and qualitative
	Measurement number and moments	1, during the cross-sectional study survey	5, once per week for the duration of the intervention	1, during the postintervention questionnaire completed directly after the intervention
	Survey language	English	English	Dutch
	Studied health behaviors	Smoking and PA^a^	Smoking and PA	Smoking
	Future-self intervention	Participants (N=144) thought about completing digital verbal and visual future-self tasks regarding their desired and undesired smoking- and PA-related future selves	Participants (N=447) completed 1 to 3 digital verbal or visual future-self tasks regarding their desired and undesired smoking- and PA-related future selves	Participants randomized to the intervention condition (n=87) completed both a digital verbal and visual future-self task regarding their desired and undesired smoking-related future selves
**Outcomes reflecting behavioral, cognitive, and affective user experiences**				
	Behavioral experiences	—^b^	Quantitative: — Qualitative: how future-self tasks were approached, done, or experienced^c^	Quantitative: time spent on future-self tasks Qualitative: what it was like to perform the future-self tasks^d^
	Cognitive experiences	Quantitative: anticipated difficulty of future-self tasks and anticipated completion time of future-self tasks Qualitative: what was anticipated to make tasks more difficult or easier than others	Quantitative: mental effort deployed on future-self tasks Qualitative: how future-self tasks were approached, done, or experienced^c^	Quantitative: experienced difficulty of future-self tasks Qualitative: what it was like to perform the future-self tasks^d^
	Affective experiences	—	Quantitative: — Qualitative: how future-self tasks were approached, done, or experienced^c^	Quantitative: emotional response to future-self tasks Qualitative: what it was like to perform the future-self tasks^d^

^a^PA: physical activity.

^b^Not applicable.

^c^The “do” part of the question was expected to probe participants to share their behavioral experiences with the future-self tasks, the “approach” part of the question was expected to probe participants to share their behavioral and cognitive experiences, and the “experience” part of the question was expected to probe participants to share their affective experiences.

^d^This question was expected to probe participants to share their behavioral, cognitive, and affective experiences with the future-self tasks. Deductive coding during data analysis allowed for the determination of which answer informed which experiential dimension.

### Data Analysis

For studies 1, 2, and 3, quantitative analyses were conducted in SPSS Statistics for Windows (version 29; IBM Corp), with statistical significance set at *P*<.05 (2-tailed). Bonferroni corrections were applied for multiple testing [51]. Effect sizes were interpreted according to the Cohen *d* (small≥0.2, medium≥0.5, and large≥0.8) for *t* tests and the Cohen *f* (small≥0.1, medium≥0.25, and large≥0.4) for ANOVAs [52]. The syntaxes are publicly available [53].

Qualitative survey data were analyzed in Microsoft Excel using qualitative content analysis principles [54]. Two researchers (KMP and a trained psychology master student) independently familiarized themselves with the responses, developed initial inductive codes, compared these, and created a coding tree. Participant responses were then coded jointly, and the coding trees were refined as needed. One researcher (KMP) subsequently created a matrix indexing all codes and counted their frequency for a weighted data representation. Finally, the findings were categorized into behavioral, cognitive, or affective experiences with future-self tasks. Specifications are detailed in the respective Data Analysis sections. Reported coding frequencies indicate how often the code appeared, not the number of participants to whom it applied.

## Study 1: Cognitive Experiences With a PA- and Smoking-Related FSI

In study 1, quantitative and qualitative survey data from the original online cross-sectional observational study [55] were used to explore cognitive experiences with a smoking- and PA-related FSI. Additional information regarding the original study can be found in the preregistration on the Open Science Framework [56], and the data related to this study are openly accessible elsewhere [57].

### Methods

#### Participants

A total of 144 participants were recruited via the online recruitment platform Prolific [58] between September 2022 and November 2022. Participants were from countries that belonged to the Organisation for Economic Co-operation and Development, excluding Turkey, Lithuania, Colombia, and Costa Rica but including South Africa [59]. Inclusion criteria required being aged 18 years, fluency in English, daily smoking, and an intention to quit within 30 days to 6 months. The original study was primarily focused on smoking cessation, which is why an inclusion criterion was formulated regarding smoking but not PA. Those familiar with similar tasks from a previous study [60] were excluded. Table 1 provides the participant characteristics.

#### Procedure

In total, 2 online surveys were administered using Qualtrics XM (Qualtrics International Inc) [61]. Interested Prolific members provided digital informed consent and completed a screening survey. Upon meeting the inclusion criteria, participants proceeded to the study survey, which included instructions for 44 different tasks that either aimed to aid smoking cessation or PA enhancement or could be beneficial to either behavior. This multistudy report focuses only on the 8 tasks targeted at one’s smoking- or PA-related future self, involving writing about or searching images describing the desired or undesired future self. Detailed instructions are provided in Multimedia Appendix 1 [55,60]. Participants were presented with all 44 preparatory tasks in a random order and provided feedback, which required approximately 40 minutes.

#### Ethical Considerations

Ethics approval for this study was granted by the Human Research Ethics Committee of Delft University of Technology (letter of approval 2338). Digital consent from participants in the original study enabled secondary analyses without additional approval. Participant data were deidentified for analysis to ensure confidentiality and privacy. Participants were compensated with £6.00 (US $7.69) per hour following Prolific regulations [62].

#### Measures

##### Personal Characteristics

These were collected from Prolific participant profiles and the screening survey and used to describe the participants. Variables included *age* (derived from year of birth), *gender* (male, including transgender male; female, including transgender female; or other), and highest completed educational level. The latter was used as an indicator of *socioeconomic position* [63] and recoded according to the International Standard Classification of Education [50]. *Smoking frequency* was assessed using the following question: “How often do you smoke tobacco products?” Participants indicating not smoking daily were informed that they could not continue taking part in the study. One question ascertained participants’ *weekly exercise* (ie, “How often do you engage in physical exercise per week?”).

##### Cognitive Experiences

In total, 3 items from the study survey were used to explore cognitive experiences with the FSI. Participants rated the *anticipated* *difficulty* of the 8 future-self tasks on a scale in which −5=*very difficult*, 0=*neutral*, and 5=*very easy*. Second, they provided an *estimated* *completion time* ranging from 0 to 30 minutes, indicating exact minutes if the anticipated time exceeded 30 minutes. Third, an open question, “Think of the preparatory activities you have just seen. What makes an activity more difficult than others?” was expected to probe participants to share their cognitive experiences with future-self tasks.

#### Data Analysis

After computing descriptive statistics, a 1-sample *t* test examined whether anticipated difficulty scores differed from the neutral 0 point. One- and 3-way repeated-measure (RM) ANOVAs explored differences in anticipated difficulty and anticipated completion time based on behavior (smoking vs PA), polarity (desired vs undesired future self), and modality (verbal vs visual description) of the future-self tasks. Assumptions were verified (Multimedia Appendix 2).

Qualitative responses ranged from 1 word to 3 sentences. All participant responses (N=144) were coded. The full coding tree can be found in Multimedia Appendix 3.

### Results

#### Quantitative Results

##### Overview

The main findings of study 1 are summarized in Table 3.

**Table 3:** Summary of quantitative and qualitative findings regarding behavioral, cognitive, and affective experiences with smoking- or physical activity (PA)–related future-self tasks from studies 1, 2, and 3.

		Study 1	Study 2	Study 3
**Behavioral experiences**				
	Quantitative findings	—^a^	—	Future-self tasks were completed in <7 minutes. Participants spent significantly more time on the verbal than on the visual task and on the desired- than on the undesired-future-self task.
	Qualitative findings	—	Participants successfully completed the future-self tasks and integrated them into daily life. Frequency, duration, time of day, and location were adapted to preferences and lifestyle. While the tasks were aimed at preparing for behavior change, multiple participants reported having changed their PA and smoking behavior or having formulated goals and action plans to do so.	—
**Cognitive experiences**				
	Quantitative findings	Future-self tasks were anticipated to be completed relatively easily and in <14 minutes. Verbal description of future selves was anticipated to take more time than visual description. It was anticipated that desired tasks, smoking-related tasks, and verbal descriptions would be easier than undesired tasks, PA-related tasks, and visual descriptions, respectively	Participants deployed slightly above-average effort on the future-self tasks. There were no differences in effort deployed on the future-self tasks depending on behavior, modality, or polarity.	Future-self tasks were experienced as relatively difficult. Visual description of future selves was experienced as more difficult than simply envisioning them.
	Qualitative findings	Tasks consisting of multiple components (eg, visualizing and writing) and requiring more time, effort, and mental visualization, particularly of one’s undesired future self, were anticipated to be more difficult. Opinions differed on whether desired- or undesired-future-self tasks were considered more difficult. Greater difficulty was anticipated to be linked to task unfamiliarity, motivation to perform the tasks, degree of self-honesty about the consequences of current behavior, attainability of the future selves, negative emotions, and finding it easier to take concrete steps to change behavior.	Some found the tasks difficult because of the mental visualization. Some found them easy because they were familiar with the tasks. The tasks appeared to trigger numerous cognitive processes, including contemplating the consequences of current and changed behavior; identifying role models; altering motivation to change; and mentally contrasting past, present, and future selves.	The desired-future-self task was seen as almost equally easy and difficult, and the undesired-future-self task was seen as (very) difficult, with individual variations. Unfamiliarity with envisioning one's future self was reported to hinder task completion, but familiarity did not always ease it. The desired-future-self task generally gave something to look forward to and sometimes prompted reflection about reasons for quitting smoking. The undesired-future-self task illustrated who participants aimed not to become, confronted them with its probability of happening or addiction, and sometimes decreased motivation to quit or prompted negative self-reflection. Future-self tasks seemed to prompt cognitive processes, including reflection about the consequences of smoking or being a smoker; increased motivation to quit smoking; cognitive dissonance; resistance to future-self thinking; or comparisons between future, current, or past selves.
**Affective experiences**				
	Quantitative findings	—	—	Participants generally felt happy during the desired-future-self task and sad, angry, or anxious during the undesired-future-self task.
	Qualitative findings	—	Desired-future-self tasks were reported to elicit positive emotions, whereas undesired-future-self tasks were reported to elicit negative emotions.	The desired-future-self task was experienced as enjoyable, and the undesired-future-self task was experienced as confronting and unpleasant, although experiences varied per individual.

^a^Not applicable for this study.

##### Anticipated Difficulty of Future-Self Tasks

**Figure 1:**
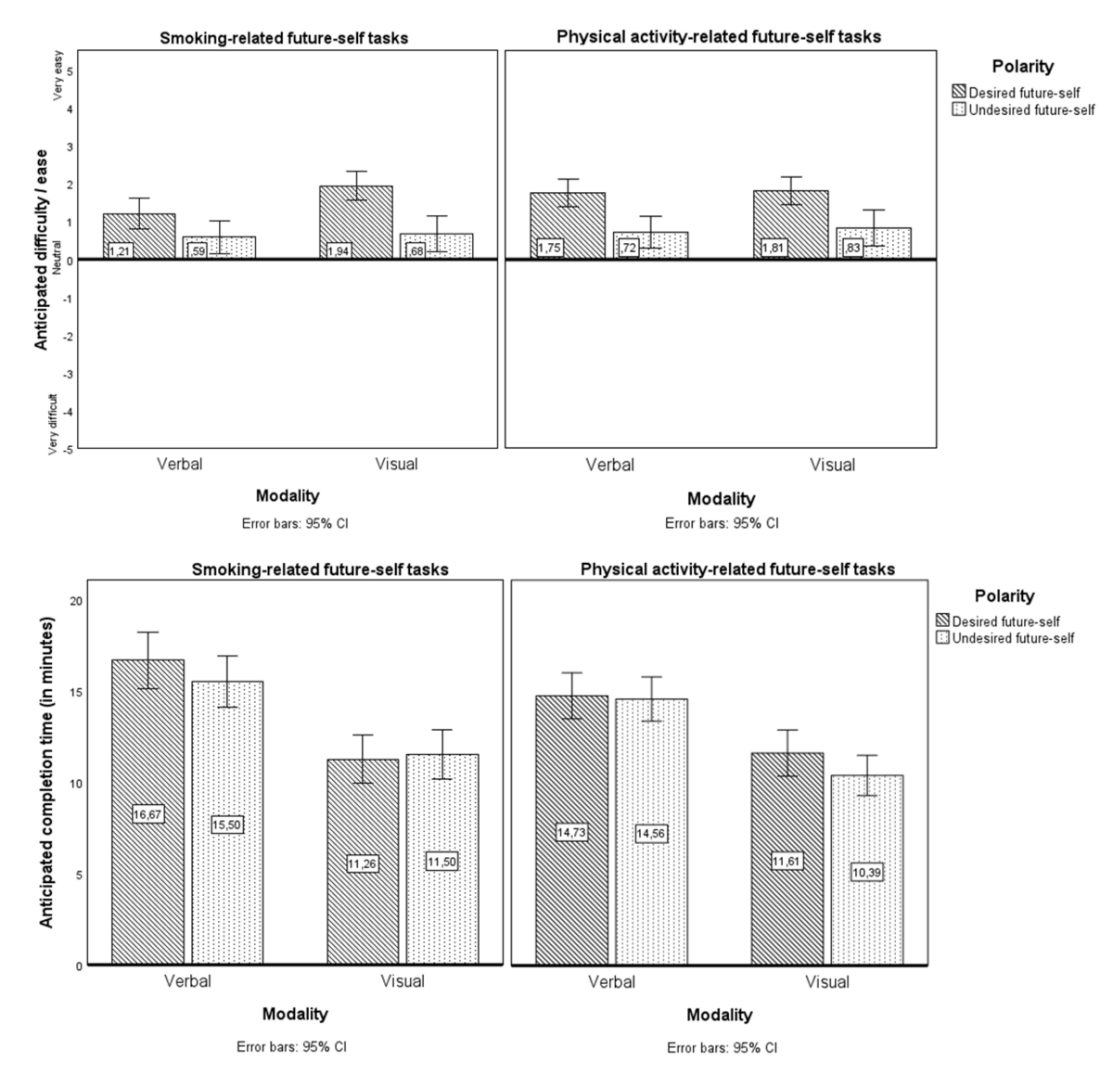
Mean anticipated difficulty scores and anticipated completion time of physical activity (PA)– and smoking-related future-self tasks by behavior (smoking or PA), polarity (desired or undesired future self), and modality (verbal or visual) in study 1 (N=144).

Figure 1 shows mean anticipated ease by behavior, modality, and polarity. A 1-sample *t* test showed that anticipated difficulty scores significantly differed from the neutral 0 point, meaning that future-self tasks were anticipated to be relatively easy to complete (mean 1.19, SD 1.75; *t*_143_*=*8.20; *P*<.001; Cohen *d=*0.68). RM ANOVAs assessed differences in anticipated difficulty based on behavior, polarity, and modality. There was a significant 3-way interaction among behavior, polarity, and modality on anticipated difficulty of the future-self tasks (*F*_1,143_=4.26; *P=*.04; η_p_^2^=0.029). Follow-up ANOVAs revealed no significant 2-way interactions or main effects of behavior and modality (lowest *P*>.01). However, they revealed significant main effects of polarity. Specifically, desired-future-self tasks were anticipated to be easier than undesired-future-self tasks for both smoking (*F*_1,143_=24.16; *P*<.001; η_p_^2^=0.145) and PA (*F*_1,143_=33.90; *P*<.001; η_p_^2^=0.192) and when asked to describe the future self verbally (*F*_1,143_=23.93; *P*<.001; η_p_^2^=0.143) and visually (*F*_1,143_=30.85; *P*<.001; η_p_^2^=0.177).

##### Anticipated Completion Time for Future-Self Tasks

Figure 1 shows mean anticipated completion time by behavior, modality, and polarity. Participants estimated that it would take, on average, 13.28 (SD 5.79) minutes to complete future-self tasks. RM ANOVAs assessed differences in anticipated completion time based on behavior, polarity, and modality. There was no statistically significant 3-way (*F*_1,130_=3.54; *P=*.06; η_p_^2^=0.027) or 2-way interaction for anticipated completion time (*P*>.05 in all cases), but there were significant main effects of behavior (*F*_1,130_=11.30; *P=*.001; η_p_^2^=0.080) and modality (*F*_1,130_=78.66; *P*<.001; η_p_^2^=0.377). Specifically, the smoking-related future-self task was anticipated to take more time to complete than the PA-related one (∆=0.91, 95% CI 0.38-1.45). In addition, verbal description of future selves was anticipated to take more time than visual description (∆=4.17, 95% CI 3.24-5.11).

#### Qualitative Results

##### Cognitive Experiences With Future-Self Tasks

Qualitative analysis indicated that tasks were anticipated to be more difficult if they involved mental visualization (25/144, 17.4%), cognitive effort (24/144, 16.7%), more time (19/144, 13.2%), physical effort (14/144, 9.7%), or multiple components (eg, both visualizing and writing about one’s future self; 4/144, 2.8%). Six reasons emerged for why visualization tasks were anticipated to be difficult: (1) unfamiliarity with mental visualization (3/144, 2.1%; *“*Visualization is a concept that is foreign to some people. A course is needed” [female participant; aged 64 years]), (2) motivation to perform the tasks or change behavior (3/144, 2.1%), (3) willingness to be honest about (the consequences of) current behavior (3/144, 2.1%), (4) perceived attainability of the future self (2/144, 2.1%), (5) expectation of a negative emotional response (2/144, 2.1%), and (6) the challenge of visualizing compared to taking concrete actions to change the behavior (2/144, 2.1%). In total, 0.7% (1/144) of the participants anticipated envisioning a positive future to be easier, whereas 2.1% (3/144) anticipated the undesired-future-self task to be easier:

For me, thinking about...positives would be difficult, as I don’t often praise myself or congratulate myself or anything like that.

Female participant; aged 50 years

### Discussion

Regarding cognitive experiences, quantitative results from study 1 showed that future-self tasks were anticipated to be completed relatively easily and in less than 14 minutes, suggesting FSIs can be anticipated to be time efficient and feasible. Desired-future-self tasks were perceived as easier than undesired-future-self tasks. Furthermore, smoking-related future-self tasks and verbal descriptions of future selves were estimated to take more time than PA-related tasks and visual descriptions. These results imply differences in experiences between specific future-self tasks.

Qualitative results indicated that tasks requiring mental visualization, cognitive or physical effort, or more time or tasks having multiple components (eg, visualizing and writing) were anticipated to be difficult. Visualization tasks were seen as challenging due to factors such as unfamiliarity, motivation, and anticipated negative emotional responses. Views varied on whether desired- or undesired-future-self tasks were more challenging in these respects. These findings suggest that, while FSIs are generally considered fairly easy, the mental visualization aspect is viewed as challenging.

## Study 2: Behavioral and Cognitive Experiences With PA- or Smoking-Related FSIs

In study 2, quantitative and qualitative survey data from the original online longitudinal observational study [55] were used to explore behavioral; cognitive; and, where possible, affective experiences with a smoking- or PA-related FSI. Additional information regarding the original study can be found in the preregistration on the Open Science Framework [64], and the data underlying this study have been published elsewhere [53].

### Methods

#### Participants

Participants were 52.5% (447/852) of the included individuals, who completed future-self tasks in the original study between February 2024 and March 2024. Inclusion criteria were being aged 18 years, fluency in English, daily smoking or vaping, intention to quit within 30 days to 6 months, not taking part in a smoking or vaping cessation intervention, and not being familiar with similar tasks from previous studies [65,66]. Participants failing attention checks integrated into the survey or not completing tasks within 2 days of having received the invitation were excluded. Dropouts were replaced until the budget of approximately €5000 (US $5475.65) was spent. The personal characteristics of study 2 participants are presented in Table 1.

#### Procedure

Prolific members who provided digital informed consent and met the inclusion criteria completed a baseline survey via Qualtrics XM, including questions about demographic characteristics. The intervention consisted of up to 5 conversational sessions of 6 to 8 minutes spaced 3 to 5 days apart with coach Kai, a text-based chatbot [67]. During these conversation sessions, participants received instructions for 1 task randomly selected from 37 tasks meant to aid smoking cessation and PA enhancement, of which 8 (22%) were future-self tasks. The tasks were a subset of those in study 1, with more detailed instructions based on the work by Albers et al [60] (Multimedia Appendix 1). In sessions 2 to 5, coach Kai asked questions about the most recent task [68].

#### Ethical Considerations

Ethics approval was granted by the Human Research Ethics Committee of Delft University of Technology (letter of approval 3683). Digital consent from participants in the original study enabled secondary analyses without additional approval. Participant data were deidentified for analysis to ensure confidentiality and privacy. Participants were compensated with £6.00 (US $7.69) per hour following Prolific regulations [62].

#### Measures

##### Personal Characteristics

The same variables as in study 1 were collected from Prolific profiles and the baseline survey with the exception of *smoking or vaping frequency*, which were combined into 1 variable. For vaping, the question was adapted to “How often do you vape?”

##### Cognitive Experiences

In total, 2 survey items administered at the start of each conversational session with coach Kai were used to explore cognitive experiences with the future-self tasks. In conversational sessions 2 to 5, coach Kai asked participants to rate their *effort* on the assigned future-self task from 0=*nothing* to 10=*extremely strong*. This served as an indication of mental effort. Participants then answered an open question, “How did you approach, do, or experience your assigned activity?” requiring a response of at least 20 characters. After providing a response, participants were asked by coach Kai whether they wanted to add something to or modify their response. The *approach* part of the question was expected to probe participants to share their cognitive experiences with future-self tasks.

##### Behavioral and Affective Experiences

The *do* and *approach* parts of a question “How did you approach, do, or experience your assigned task?” were expected to probe participants to share their behavioral experiences with future-self tasks, whereas the *experience* part was expected to probe them to share affective experiences.

#### Data Analysis

Descriptive statistics were computed. Two 1-sample *t* tests examined whether mean effort on the 8 future-self tasks differed from (1) the midpoint of the scale (ie, 5) and (2) mean effort on the other 29 preparatory tasks. The effort score of 0.2% (1/447) of the participants was adjusted from 0 to 5 following self-report in the free-text response field that they had erroneously selected 0. Three-way and follow-up 1-way ANOVAs explored differences in effort based on behavior (smoking, PA, or both), polarity (desired or undesired future self or both), and modality (verbal or visual description or both) of the future-self tasks. Assumptions were verified (Multimedia Appendix 2).

Free-text survey answers were coded until no new codes emerged. This resulted in 17.6% (103/586) of free-text responses from 21.3% (95/447) of the participants. Responses ranged from 1 word to 13 sentences. The full coding tree can be found in Multimedia Appendix 3.

### Results

The main findings of study 2 are summarized in Table 3.

#### Quantitative Results: Effort Deployed on Future-Self Tasks

**Figure 2:**
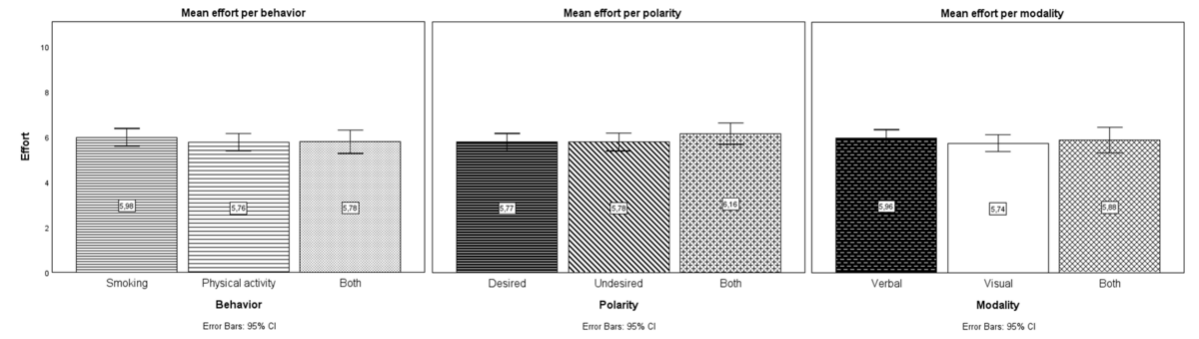
Mean effort deployed on physical activity (PA)– or smoking-related future-self tasks by behavior (smoking, PA, or both), polarity (desired or undesired future self or both), and modality (verbal or visual or both) in study 2 (N=447).

Participants completed between 1 and 3 future-self tasks. One-sample *t* tests showed that participants deployed significantly above-average amounts of effort on future-self tasks (mean 5.85, SD 2.56; *t*_446_*=*7.02; *P*<.001; Cohen *d=*0.33), akin to the effort deployed on the other preparatory tasks from the same study (*t*_446_=0.92; *P*=.36; Cohen *d=*0.04). Figure 2 shows mean effort by behavior, polarity, and modality. A 3-way ANOVA assessed possible differences in effort based on behavior, polarity, and modality. There was no significant interaction between behavior, polarity, or modality and effort (*F*_1, 424_=1.04; *P=*.39; η_p_^2^=0.010) and no significant main effects of behavior (*F*_1, 444_=0.38; *P=*.69; η_p_^2^=0.002), modality (*F*_1,444_=0.80; *P=*.45; η_p_^2^=0.004), or polarity (*F*_1,444_=0.37; *P=*.69; η_p_^2^=0.002).

#### Qualitative Results

##### Behavioral Experiences With Future-Self Tasks

Participants reported success in writing about their future selves (2/95, 2%) or finding an image representing them (13/95, 14%):

I found a picture of a graveyard, and imagined being laid to rest there earlier than I might be.

Male participant; aged 59 years

Several participants reported on the frequency (eg, 1-2 times a day or when craving a cigarette), duration (eg, needing several days to form a clear mental image [male participant; aged 31 years]), time of day (eg, before sleep [male participant; aged 23 years]), or conditions (eg, in a relaxed, quiet, distraction-free environment, 3/95, 3%) required for completing their future-self tasks. In total, 3% (3/95) of the participants were unable to complete the tasks due to forgetting, illness, or parental duties. Overall, participants adapted the future-self tasks to their preferences and lifestyles and integrated them into their lives.

The future-self tasks were reported to have various behavioral outcomes. A total of 15% (14/95) of the participants saw instructions as encouraging them to quit smoking or become more physically active:

I just made an effort to go for a short walk every day after work to destress.

Female participant; aged 33 years

In total, 2% (2/95) of the participants searched for information on smoking’s health effects, and others felt invited to formulate goals (11/95, 12%) or an action plan (5/95, 5%) regarding smoking or PA. A total of 8% (8/95) reported the task to be ineffective in changing their PA or smoking behavior:

I found that this task to help quit vaping was ineffective for me as I found it difficult to visualize myself in the future.

Male participant; aged 59 years

##### Cognitive Experiences With Future-Self Tasks

There was quite some variation in how participants experienced future-self tasks. In total, 2% (2/95) found them easy, whereas 5% (5/95) found them difficult. Reported reasons for seeing them as difficult included the inability to produce a clear mental image (5/95, 5%), refusal to think about (1/95, 1%) or identify with (1/95, 1%) an undesired future self, not perceiving the need to quit vaping (1/95, 1%), or addiction standing in the way of visualizing a future without smoking (1/95, 1%).

The future-self tasks were reported to trigger various cognitive processes. The most common was thinking about the consequences of current (14/95, 15%) or changed behavior (9/95, 9%):

I took my time to think about the negative things about vaping and be clear about the type of person I don’t want to become.

Male participant; aged 42 years

The second most common cognitive process was facing one’s fears (eg, of disease, premature death, or being a bad romantic partner and example to children, 6/95, 6%). The third most common was identifying positive (3/95, 3%) or negative (3/95, 3%) role models representative of desired or undesired future selves. The fourth most common was comparing desired and undesired future selves (1/95, 1%), desired future or past selves with the undesired current self (1/95, 1%), or past with desired future selves (5/95, 5%; eg, “I looked at pictures of myself when I was at my heaviest weight. I was close to 200 lbs, smoking, feeling like garbage. I don’t EVER want to become that person again” [female participant; aged 57 years]). The fifth most common was increased or decreased motivation for changing behavior following the tasks (1/95, 1%).

##### Affective Experiences With Future-Self Tasks

Tasks related to the undesired future self were experienced as confronting (6/95, 6%), anxiety inducing (2/95, 2%), worrisome (1/95, 1%), or saddening (1/95, 1%). In contrast, desired-future-self tasks felt inspiring (1/95, 1%) and provided something to look forward to (3/95, 3%).

### Discussion

Regarding behavioral experiences, qualitative findings from study 2 indicated that participants successfully integrated future-self tasks into their lives by adjusting frequency, duration, timing, and location to suit their lifestyles and preferences. This aligns with study 1, suggesting that FSIs are feasible for smokers intending to quit and for varying levels of PA. Although the tasks aimed to help prepare for behavior change, numerous participants actually changed their PA and smoking behaviors or formulated goals and action plans to do so, suggesting that the tasks were stimulating enough to prompt action.

Regarding cognitive experiences, quantitative findings showed that participants exerted above-average effort on future-self tasks regardless of behavior, polarity, or modality. This implies that tasks were appropriately challenging. Similar to study 1, mental visualization was seen as complicating the tasks, a result formally assessed in study 3. Future-self tasks were perceived as easier with increased familiarity. They also appeared to stimulate various cognitive processes, suggesting that repeated practice may further enhance engagement.

Regarding affective experiences, future-self tasks elicited contrasting emotional responses—negative for undesired-future-self tasks and positive for desired-future-self tasks—highlighting the importance of considering emotional engagement with FSIs.

## Study 3: Behavioral, Cognitive, and Affective Experiences With a Smoking-Related FSI

In study 3, quantitative and qualitative survey data from the original online longitudinal experimental study were used to explore behavioral, cognitive, and affective experiences with a smoking-related FSI. Additional information regarding the methods and results of the original study is reported elsewhere [20,69], and the underlying data are openly accessible [70].

### Methods

#### Participants

Participants were 43.3% (87/201) of the individuals included in the original study (ie, those who completed the FSI). Recruited in the Netherlands and Dutch-speaking part of Belgium between July 2017 and October 2018, they were sourced through various media channels (eg, participation in previous research and social media—the full list of recruitment methods is available elsewhere [20]). Inclusion criteria were being aged ≥18 years, smoking daily, and intending to quit smoking sometime in the future. Participant characteristics are presented in Table 1.

#### Procedure

Data were collected using Qualtrics XM. After providing digital informed consent, participants completed the baseline survey and were randomized 1:1 to the intervention or waitlist control condition. Intervention participants imagined a future in which they successfully quit smoking, wrote about this future self (verbal task), and uploaded images describing it (visual task). Afterward, they completed a postintervention survey, which took approximately 20 minutes. Control participants completed similar tasks about washing their hands more often.

#### Ethical Considerations

Ethics approval was granted by the Research Ethics Committee of Leiden University’s Institute of Psychology (CEP17-0505/192). Digital consent from participants in the original study enabled secondary analyses without additional approval. Participant data were deidentified for analysis to ensure confidentiality and privacy. In total, 2 gift coupons of €100 (US $109.51) and 6 coupons of €50 (US $54.76) were randomly distributed among participants who completed the study.

#### Measures

##### Personal Characteristics

Participants indicated their *gender*, *birth year*, and *cigarette consumption*, which was recoded to match smoking frequency in studies 1 and 2. Educational level was used as an indicator of *socioeconomic position* (as in the studies by Penfornis et al [20] and Meijer et al [71]), measured using answer options from *no education* to *university* and recoded according to the International Standard Classification of Education [50].

##### Behavioral Experiences

This was measured through *time spent* on each future-self task (in minutes), extracted from Qualtrics, and 2 open questions about *experiences with the desired- and undesired-future-self tasks* (ie, “What was it like for you to perform the tasks about yourself in a future when you have [successfully quit/continued] smoking?”). The 2 open questions were expected to probe participants to share their behavioral experiences with future-self tasks. There was no minimum or maximum length for answers.

##### Cognitive Experiences

A total of 8 items assessed the *experienced difficulty* of different elements of future-self tasks (ie, “How difficult did you find it to do the following things: [visualize/search for images/write a text/write keywords] describing yourself [as successfully quit smoker/continued smoker]?”), with answer options 1=*very difficult* to 7=*very easy*. The variable was recoded so that a higher score indicated greater difficulty. Scores for writing a short text and writing keywords were averaged to create 1 verbal difficulty score. The 2 open questions about *experiences with the desired- and undesired-future-self tasks* were expected to probe participants to share their cognitive experiences with future-self tasks.

##### Affective Experiences

In total, 8 items ascertained *emotional responses* to future-self tasks, asking participants to what extent they felt happy, scared, sad, or angry when imagining a future in which they successfully quit or continued smoking, rated from 1=*totally disagree* to 5=*totally agree*. An average score was computed for each emotion. The 2 open questions about *experiences with the desired- and undesired-future-self tasks* were expected to probe participants to share their affective experiences with future-self tasks.

#### Data Analysis

Descriptive statistics were computed, and a 1-sample *t* test examined differences from 3.5, the midpoint of the scale, in the average experienced difficulty across the 2 future-self tasks. RM ANOVAs explored differences in (1) time spent based on polarity (desired vs undesired future self) and modality (verbal vs visual description of the future self) and (2) experienced difficulty based on polarity and future-self task element (ie, mental visualization vs verbal vs visual description of the future self). A multivariate ANOVA ascertained differences in the strength of the 4 emotions (happy, scared, sad, and angry) based on polarity. Assumptions were verified (Multimedia Appendix 2).

Answers to both open questions—which ranged from 1 word to 3 sentences—were coded for all participants who provided one (83/87, 95%). In total, 6% (5/83) of the participants provided identical answers for both future-self tasks, which were counted and interpreted only on first occurrence. A total of 6 answers of only a few characters in length or irrelevant for describing the experience (eg, “looking in the future”) were excluded from the analysis. The full coding tree can be found in Multimedia Appendix 3.

### Results

The main findings are summarized in Table 3.

#### Quantitative Results

##### Time Spent on Future-Self Tasks

**Figure 3:**
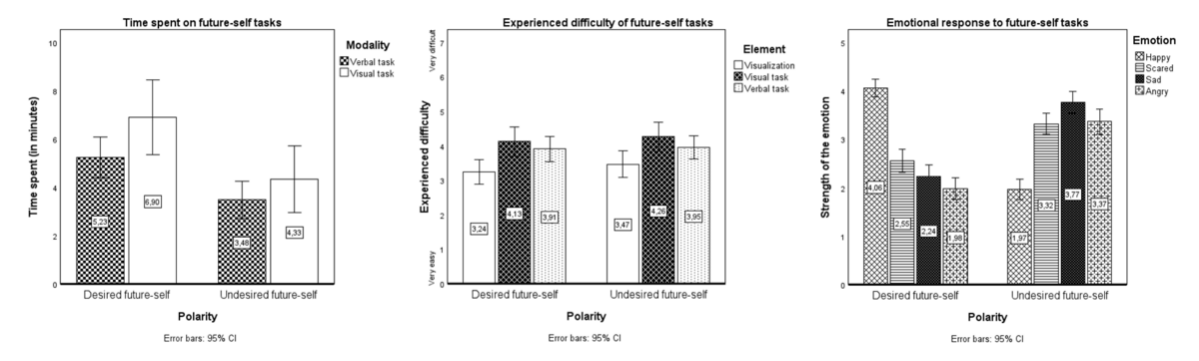
Mean experienced difficulty with, time spent on, and emotional response to smoking-related future-self tasks by polarity (desired or undesired future self) and modality (visualizing the future self, describing it verbally, or describing it visually) in study 3 (N=87).

Future-self tasks were completed in an average of 6.64 (SD 8.30) minutes. Figure 3 shows mean time spent by polarity and modality. An RM ANOVA assessed differences in time spent based on polarity and modality. There was no significant interaction effect (*F*_1,81_=0.74; *P=*.39; η_p_^2^=0.009), but there were significant main effects of polarity (*F*_1,81_=23.81; *P*<.001; η_p_^2^=0.227) and modality (*F*_1,81_=5.18; *P*=.03; η_p_^2^=0.060) on time spent. Specifically, participants spent significantly more time on the verbal than on the visual task (∆=1.26, 95% CI 0.16-2.36) and on the desired- than on the undesired-future-self task (∆=2.16, 95% CI 1.28-3.04).

##### Experienced Difficulty of Future-Self Tasks

Future-self tasks were experienced as relatively difficult (mean 3.83, SD 1.02; *t*_86_*=*3.00; *P*=.002; Cohen *d=*0.32). Figure 3 shows mean experienced difficulty by polarity and task element. An RM ANOVA assessed differences in experienced difficulty based on polarity and task element. There was no significant interaction between polarity and element regarding experienced difficulty (*F*_2,85_=0.23; *P=*.80; η_p_^2^=0.005). There was a significant main effect of element (*F*_2,85_=8.32; *P*<.001; η_p_^2^=0.134). Specifically, searching for images summarizing the future selves was experienced as significantly more difficult than merely envisioning the future self (∆=−0.84, 95% CI −1.30 to −0.30).

##### Emotional Response to Future-Self Tasks

Figure 3 shows mean strength of each emotion by polarity. A multivariate ANOVA ascertained differences in the strength of the 4 emotions based on polarity. There was a significant main effect of polarity (*F*_4,83_=35.38; *P*<.001; η_p_^2^=0.630). Specifically, the desired-future-self task elicited significantly more happiness than the undesired-future-self task (*F*_1,86_=141.58; *P*<.001; η_p_^2^=0.622). The undesired-future-self task elicited significantly more anger (*F*_1,86_=61.14; *P*<.001; η_p_^2^=0.416), sadness (*F*_1,86_=66.31; *P*<.001; η_p_^2^=0.435), and anxiety (*F*_1,86_=21.60; *P*<.001; η_p_^2^=0.201).

#### Qualitative Results

##### Behavioral Experiences With Future-Self Tasks

Only 2% (2/83) of the responses informed about behavioral experiences with the tasks. Specifically, one participant printed and kept the images describing his future selves, and another mentioned finding the task to be difficult to translate into action.

##### Cognitive Experiences With Future-Self Tasks

Nearly equal numbers of participants found the desired-future-self task easy (11/83, 13%) or difficult (8/83, 10%), with some describing it as inspiring (3/83, 4%), interesting (3/83, 4%), or important (2/83, 2%). Individual cases found it empowering, insightful, and peculiar. The undesired-future-self task was mostly considered (very) difficult (17/83, 20%). A few participants (2/83, 2%) found it easy, and individual cases found it peculiar, important, interesting, useful, and inspiring. In total, 1% (1/83) of the participants found both tasks equally easy, 2% (2/83) found the undesired-future-self task easier, and 5% (4/83) found the desired-future-self task easier due to difficulties imagining a negative, unwanted outcome.

Familiarity with envisioning one’s future self seemed impactful for task completion. Some participants (2/83, 2%) mentioned that it was their first time considering their future self, whereas others had done so once (1/83, 1%) or repeatedly (3/83, 4%) before. In total, 2% (2/83) of the participants, who were first timers, found the task difficult or “peculiar. I suddenly had to think about what a future without smoking actually means” (female participant; aged 28 years). Among those with previous experience, one participant reported no effect, another participant visualized a clearer future self this time around, and 2 others found the task easy or still difficult.

The desired future self provided something to look forward to (7/83, 8%), although some (3/83, 4%) found it unattainable. It fostered positive perceptions of the future or future self (2/83, 2%) and, in individual cases, prompted reflection on reasons to quit smoking and attitudes toward smoking or had little effect. The undesired-future-self task made clear who participants did not want to become (10/83, 12%):

I don’t want to become the person I outlined myself—surely a right-thinking person doesn’t poison herself!

Female participant; aged 35 years

It confronted some with their addiction (2/83, 2%) or its likelihood (2/83, 2%), decreased motivation to quit smoking (1/83, 1%), or felt like “you have to tell bad things about yourself” (male participant; aged 75 years).

Participants’ responses suggest that future-self tasks triggered various cognitive processes, including reflection on smoking consequences (10/83, 12%), cognitive dissonance (8/83, 10%), increased motivation to quit smoking (7/83, 8%), resistance to future-self thinking (3/83, 4%), comparing current and future selves (5/83, 6%) or future and past selves (2/83, 2%), and considering the meaning of being a smoker (2/83, 2%).

Preferences regarding the order of the future-self tasks were mixed. One participant preferred the desired-future-self task first for its pleasantness, whereas another favored the undesired-future-self task first to end positively:

Now the smoker is in my head instead of the nonsmoker.

Male participant; aged 53 years

##### Affective Experiences With Future-Self Tasks

A total of 17 adjectives were used to describe affective experiences with future-self tasks, both positive and negative. The desired-future-self task was most often called *enjoyable* (21/83, 25%). It was also described as positive (6/83, 7%), confronting (3/83, 4%), unpleasant (1/83, 1%), and depressing (1/83, 1%).

The undesired-future-self task was primarily described as confronting (21/83, 25%), unpleasant (10/83, 12%), depressing (5/83, 6%), negative (6/83, 7%), enlightening (3/83, 4%), anxiety inducing (3/83, 4%), and uncomfortable (2/83, 2%). Some described it as frustrating, humiliating, neutral, positive, and triggering insecurity. Notably, 2% (2/83) of the participants found the task enjoyable.

### Discussion

Regarding behavioral experiences, quantitative findings from study 3 showed that future-self tasks were completed in less than 7 minutes, with more time devoted to the desired-future-self and verbal tasks. This indicates that FSIs can be time efficient.

Concerning cognitive experiences, quantitative results indicated that future-self tasks were considered relatively difficult, with visual description of future selves being harder than visualization. Qualitative results revealed variations in experienced difficulty—the desired-future-self task was viewed as both easy and difficult, whereas the undesired-future-self task was mostly seen as difficult. Furthermore, as observed in studies 1 and 2, unfamiliarity with envisioning one’s future self seemed to hinder task performance, although familiarity did not always ease it. Finally, task order preferences varied. These findings highlight the importance of considering differences in individual experiences with FSIs.

In line with the findings of studies 1 and 2, future-self tasks appeared to trigger numerous cognitive processes, further hinting at FSIs having the capacity to prompt action. Coherent with its concept, the desired future self generally provided something to look forward to and sometimes led to reflections on quitting smoking. Conversely, the undesired future self served as something to avoid, was seen as confronting, and sometimes decreased motivation to quit or prompted negative self-reflection.

Regarding affective experiences, quantitative results showed that participants mostly felt happy during the desired-future-self task and sad, angry, or anxious during the undesired-future-self task. Qualitative results supported this, with the desired-future-self task described as enjoyable and the undesired-future-self task described as confronting and unpleasant, although with individual variations. These experiences are understandable given the concept of the future-self tasks and suggest that they had the intended effect.

## General Discussion

### Principal Findings

#### Overview

This multistudy report involved various smoking- and PA-related FSIs from multiple empirical studies comprising a large number of participants. It is the first to explore user experiences with smoking- and PA-related FSIs, triangulating behavioral, cognitive, and affective dimensions. Regarding behavioral experiences, participants generally reported successful and timely integration of FSIs into daily life, with some noting changes in PA or smoking behavior despite the tasks being preparatory. Regarding cognitive experiences, a moderate effort was exerted, with task difficulty varying by individual and task, yet future-self tasks consistently prompted cognitive processes supporting behavior change. Regarding affective experiences, desired-future-self tasks were generally seen as enjoyable and happiness inducing, whereas undesired-future-self tasks were perceived as confronting, unpleasant, and evoking negative emotions. The findings of all 3 studies are summarized in Table 3.

#### Behavioral Experiences With FSIs

Study 3 reflected that participants successfully and quickly completed digital FSIs and integrated them into their lives. With a completion time of less than 7 minutes—much less than the 15 minutes anticipated in study 1 and 14 to 20 minutes reported in previous studies [37,38]—these interventions seem time efficient. Differences in completion time in this study compared to previous ones may be due to the digital format allowing for more freedom over exercise length. The discrepancy with study 1’s estimate may stem from difficulties estimating time requirements without completing the intervention.

The successful integration of FSIs into daily life suggests feasibility and acceptability. Participants did not provide negative comments about the digital format, which further supports the feasibility and acceptability of digital implementation. Digital implementation, in turn, optimizes human and monetary resources for smoking cessation and PA promotion and allows for delivering support anywhere, anytime [8].

Studies 1 and 3 provided additional insights into future-self task completion time. Study 3 revealed that verbal descriptions took longer than visual ones, consistent with study 1’s expectations. This may be because visually describing the future self extends the process of producing mental images, partly relying on the same visual cognitive processes [69,72], whereas verbal future-self tasks, which rely on different cognitive processes, would understandably take longer. Alternatively, participants generally used existing images for visual descriptions, which is quicker than creating written materials. Future research could validate and shed light on the origin of this difference. Study 3 also showed that desired-future-self tasks took longer than undesired-future-self ones, possibly because participants were more inclined to invest time in considering and describing a positive future self. Therefore, future research and FSI designs could explore how to optimize task formats to balance cognitive engagement, practical feasibility, and impact.

One study 2 participant attempted to quit smoking, and multiple participants increased their PA after the future-self tasks, suggesting that FSIs can be effective in influencing behavior. These findings echo previous successes of FSIs in influencing smoking and PA behaviors [31,33-38] and support the continued use of these interventions to promote change in these health behaviors. In contrast, no behavior changes were observed in study 3, possibly because study 2 participants were explicitly told that FSIs might help quit smoking—a hypothesis for future research. Reported changes mainly involved increased PA, possibly because the PA-related tasks in study 2 presented PA as a potential facilitator of smoking cessation, empowering participants to increase their PA as a first step. Alternatively, as the participants were daily smokers and, in most cases, regular exercisers, smoking and PA were likely anchored in their identity. We know that people prefer to act in line with their identity and will try to avoid behaviors that do not align with or threaten it [16,73]. Thus, tasks aiming to connect nonsmoking with their identity may have felt less self-relevant or threatening, discouraging changes, whereas PA-related tasks possibly reinforced PA identity, encouraging the behavior. These findings suggest that interventions are more effective when they build on established identity or carefully frame new behaviors, such as nonsmoking, to align with or positively reinforce the individual’s current identity.

#### Cognitive Experiences With FSIs

Slightly above-average effort was exerted to complete the tasks in study 2, akin to effort deployed in comparable future-self tasks [74] and the other preparatory tasks from the study. This effort level suggests that the tasks either included fitting elements to keep individuals engaged [71] or lacked such elements, resulting in individuals not being sufficiently engaged to exert more effort. This effort level further implies that participants took the tasks seriously, possibly because they found them useful and meaningful [37,60,76,77], and may indicate tasks of appropriate difficulty and emotional engagement (see the motivational intensity theory [74,75]). No significant differences in effort were observed based on behavior, polarity, or modality, suggesting equal willingness to describe the desired or undesired future self verbally or visually regardless of whether the behavior is health compromising or health promoting. While the aforementioned findings are insightful, more research (eg, conducting in-depth interviews with users to understand their perceptions of effort) would be beneficial for unequivocal conclusions.

Across all studies, task difficulty varied depending on individuals, tasks, and quantitative versus qualitative data. More specifically, in study 1, future-self tasks were anticipated to be relatively easy, whereas in study 3, they were experienced as fairly difficult. In study 1, undesired-future-self, PA-related, and visual future-self tasks were anticipated to be harder, but in study 3, only visual tasks were perceived as more challenging. In addition, while in studies 1 and 2 participants often attributed difficulty to visualization, study 3 found it to be the easiest task component, with visual description being the most challenging. Given that study 3 actually measured difficulty directly—unlike study 1, which measured anticipated completion time—its findings could be considered more reliable. However, its qualitative results partly contradicted its quantitative findings as envisioning future selves was generally considered difficult. In summary, conclusions about future-self task difficulty are inconsistent, warranting further investigation. In total, 2 exceptions emerged. First, visual future-self tasks were generally seen as difficult, suggesting that verbal tasks may be preferable for future interventions, although more time-consuming. Second, all 3 studies found that unfamiliarity with mental visualization complicated future-self tasks, supporting the teaching [38,41] and practice of mental visualization.

Future-self tasks in studies 2 and 3 were reported to trigger cognitive processes (eg, considering the consequences of PA or smoking behavior or behavior change motivation fluctuations, which typically precede behavior change [78-81]). Sometimes, the tasks seemed to trigger mental contrasting among past, present, or future selves, a process beneficial for achieving the desired identity and avoiding the undesired one [29,82]. Coupled with behavior change reports, it seems that future-self tasks can effectively prepare participants for behavior change by connecting healthier behaviors with their identity. Future research could assess whether FSIs are more suited to change certain health behaviors and whether they have the capacity to change multiple health behaviors simultaneously [7,83].

#### Affective Experiences With FSIs

All studies showed that desired-future-self tasks were generally experienced as positive and enjoyable and associated with happiness, aligning with previous research [42,43]. Conversely, undesired-future-self tasks were reported as negative, confronting, and unpleasant and linked to feeling scared, sad, or angry, also supporting previous findings [43]. By allowing individuals to anticipate feeling what it is like to (not) be someone who engages in a certain behavior, future-self imagery can drive intentional behavior change [27,28] and sustain desire for change until the behavior becomes habitual [84]. Therefore, despite causing some measure of negative arousal, the undesired-future-self task appears crucial for motivating behavior change and should remain part of FSIs. To minimize psychological distress, strategies such as ending with desired-future-self tasks, allowing for task order choice, rehearsing steps to avoid or achieve the undesired or desired future self to boost self-efficacy [33,37,84], or arranging access to (professional) human support could be used.

### Strengths and Limitations

This multistudy report has several limitations. First, conducting secondary analyses on data from different studies with diverse original aims, interventions, and methodologies complicated the triangulation and interpretation of findings. However, combining mixed methods data across interventions and research fields provided richer insights into user experiences, which can guide hypothesis formulation in future research. Second, post hoc power analyses showed that some effects, especially interaction effects in studies 1 and 3, fell below the conventional 0.80 threshold. Hence, certain effects may have been missed, which calls for research using larger, adequately powered samples to validate the findings. Nevertheless, we systematically tested main and simple main effects across all factors, reducing the likelihood of having missed essential patterns in the data. Third, as the participants were predominantly highly educated—and, therefore, likely digitally literate and used to performing tasks requiring cognitive load—some sampling bias may have been introduced. The findings of this multistudy report may not transfer to populations with middle and lower educational levels [85], in particular people who smoke [86-88], and have to be interpreted carefully. Fourth, while all studies mostly relied on self-report measures, which are less objective than use or performance measures, such measures are commonly used in research and valued for their convenience, efficiency [89], and capacity to capture complex experiences. In addition, combining qualitative and quantitative data and exploring behavioral, cognitive, and affective aspects likely mitigated the limitations of individual studies, offering a more thorough perspective on user experiences with FSIs.

### Conclusions

The findings of this multistudy report support the future use of FSIs as a time-efficient, feasible, and acceptable method for promoting smoking cessation and PA by connecting healthier behaviors with self-identity. Experiences with FSIs did not appear to significantly differ between smoking, a health-compromising behavior, and PA, a health-promoting behavior. Digital administration seemed acceptable and is beneficial for efficient use of human and monetary resources. We encourage FSIs to include both a desired- and undesired-future-self task and train users in mental visualization. The intervention may be improved by tailoring it to individual preferences, such as choosing to start with the desired- or undesired-future-self task. The results enhance the scientific understanding of FSIs and offer guidance for designing tailored, effective, scalable, user-centered digital interventions promoting smoking cessation and PA. While the results encourage further use, inconsistencies in them highlight the need for more research to optimize FSIs, such as further assessing user experiences; the effectiveness of FSIs in influencing multiple health behaviors; and the impact of factors such as socioeconomic position, self-identity strength, or behavioral history.

## Appendix

Multimedia Appendix 1 Instructions for the future-self interventions used in studies 1, 2, and 3.

Multimedia Appendix 2 Results of assumption testing and robustness analyses.

Multimedia Appendix 3 Coding trees for qualitative analyses.

## Abbreviations

FSI: future-self intervention
PA: physical activity
RM: repeated-measure

## Data Availability

The datasets generated or analyzed during study 1 [57], study 2 [53], and study 3 [20], as well as the analysis codes for all 3 studies are available online [53].
