# Supplementary material for: User Experiences With Digital Future-Self Interventions in the Contexts of Smoking and Physical Inactivity: Mixed Methods Multistudy Exploration
Source: JMIR Form Res. 2025 Jun 20;9:e63893. doi: 10.2196/63893 (PMC12228003; doi:10.2196/63893)
Supplement: Multimedia Appendix 1 [file formative_v9i1e63893_app1.docx]

# **Supplement 1 – Instructions for the future-self interventions used in Study 1, Study 2 and Study 3**

| **Behavior Polarity Modality** | **Study 1 ^a^** | **Study 2^a^** | **Study 3^b^** |
| --- | --- | --- | --- |
|  |  |  |  |
| Smoking  Desired future-self  Verbal task | *[How difficult or easy do you think is the activity?*  *How much time do you think it takes to do the activity (in minutes)?]*  Writing about one's desired future self after quitting smoking. | Having high aspiration to quit smoking may aid in quitting successfully. Thus, after this session, I advise you to think about the person that you would like to be once you have successfully quit smoking. For example, you might want to be a "grandfather who can play football with his grandchildren" or a "nurse who can walk up the stairs to the fourth floor without getting out of breath." Write down everything that comes to your mind. | *Imagine that you have successfully quit smoking and try to visualize this. Consider what kind of person you will be and how your life will look. Why does it fit with who you want to be? What qualities do you hope to have as a non-smoker?*  *Think of yourself as someone who has successfully quit smoking. Consider how you have become closer to the person you would like to be. Also, think about how you have moved further away from the person you are afraid of becoming. Fully immerse yourself in this future vision as best as you can. Take all the time you need to imagine this and empathize with it …*  … Now describe the image you have of yourself when you have successfully quit smoking. Take about five minutes for this. Of course, you can take longer if you like. Try to keep writing continuously. Don't overthink what you write or how you write it. There are no right or wrong answers.  Try to write at least ten lines. If you want, you can write more. |
| Smoking  Desired future-self  Visual task | Looking for or taking a picture that visualizes one's desired future self after quitting smoking. | Having high aspiration to quit smoking may aid in quitting successfully. Thus, after this session, I advise you to think about the person that you would like to be once you have successfully quit smoking. For example, you might want to be a "strong woman who lives a healthy life" or a "father who is a good role model for his children." Then look for or take a picture that best captures your desired future self. Save or print this picture so that you can see it every day. | … Now find five images that fit well with what you just imagined.  These can be anything, such as personal photos, scans, drawings, images from the internet, and so on. It's important that these images align well with the future image you have of yourself when you have successfully quit smoking.  Try not to spend too much time on this again. Once you've found images that you feel fit well enough, that's good. There are no right or wrong choices. |
| Smoking  Undesired future-self  Verbal task | Writing about one's feared future self when not quitting smoking. | Having high motivation to quit smoking may aid in quitting successfully. Thus, after this session, I advise you to think about who you do NOT want to be in the future but might become if you continue to smoke. For example, you might NOT want to be a "mother who dies early of coronary heart disease as her mother did," a "husband who is frowned upon by his wife" or a "man who is dependent on a substance." Write down everything that comes to your mind. | *Think again about yourself in the future.*  *Now imagine that you have continued smoking. Try to visualize this. Consider what kind of person you will be and how your life will look. Why does it not fit with who you want to be? What qualities do you hope not to have as a persistent smoker?*  *Think of yourself as someone who has continued smoking. Consider how you have become closer to the person you are afraid of becoming. Also, think about how you have moved further away from the person you would like to be. Fully immerse yourself in this future vision as best as you can. Take all the time you need to imagine this and empathize with it …*  … Now describe the image you have of yourself if you have continued smoking.. Take about five minutes for this. Of course, you can take longer if you like. Try to keep writing continuously. Don't overthink what you write or how you write it. There are no right or wrong answers.  Try to write at least ten lines. If you want, you can write more |
| Smoking  Undesired future-self  Visual task | Looking for or taking a picture that visualizes one's feared future self when not quitting smoking. | Having high motivation to quit smoking may help to quit successfully. So, after this session, I advise you to think about who you do NOT want to be in the future but might become if you continue to smoke. For example, you might NOT want to be a "mother who dies early of coronary heart disease as her mother did," a "husband who is frowned upon by his wife" or a "man who is dependent on a substance." Then, look for or take a picture that best captures your feared future self. Save or print this picture so that you can see it every day. | … … Now find five images that fit well with what you just imagined.  These can be anything, such as personal photos, scans, drawings, images from the internet, and so on. It's important that these images align well with the future image you have of yourself when you have successfully quit smoking.  Try not to spend too much time on this again. Once you've found images that you feel fit well enough, that's good. There are no right or wrong choices. |
| Physical activity  Desired future-self  Verbal task | Writing about one's desired future self after becoming more physically active. | Quitting smoking may be easier if you become more physically active (e.g., take walks, swim, or go running). One important step for this is to have a high ambition to become more physically active. Thus, after this session, I advise you to think about the person that you would like to be once you have become more physically active. For example, you might want to be a "grandfather who can play football with his grandchildren" or a "nurse who can walk up the stairs to the fourth floor without getting out of breath." Write down everything that comes to your mind. | - |
| Physical activity  Desired future-self  Visual task | Looking for or taking a picture that visualizes one's desired future self after becoming more physically active. | Quitting smoking may be easier if you become more physically active (e.g., take walks, dance, or swim). One crucial step for this is to have high motivation to become more physically active. Thus, after this session, I advise you to think about the person that you would like to be once you have become more physically active. For example, you might want to be a "grandfather who can play football with his grandchildren" or a "nurse who can walk up the stairs to the fourth floor without getting out of breath." Then look for or take a picture that best captures your desired future self. Save or print this picture so that you can see it every day. | - |
| Physical activity  Undesired future-self  Verbal task | Writing about one's feared future self when not becoming more physically active. | It may be easier to successfully quit smoking if you become more physically active (e.g., swim, take walks, or dance). One crucial step for this is to have high determination to become more physically active. Therefore, after this session, I advise you to think about who you do NOT want to be in the future but might become if you fail to become more physically active. For example, you might NOT want to be a "mother who dies early of coronary heart disease as her father did," a "daughter who is frowned upon by her mother" or a "man who is dependent on his wife in his everyday life." Write down everything that comes to your mind. | - |
| Physical activity Undesired future-self  Visual task | Looking for or taking a picture that visualizes one's feared future self when not becoming more physically active. | It may be easier to successfully quit smoking if you become more physically active (e.g. exercise, take walks, sit less). One crucial step for this is to have high determination to become more physically active. Therefore, after this session, I advise you to think about who you do NOT want to be in the future but might become if you fail to become more physically active. For example, you might NOT want to be a "mother who dies early of coronary heart disease as her father did," a "daughter who is frowned upon by her mother" or a "man who is dependent on his wife in his everyday life." Then, look for or take a picture that best captures your feared future self. Save or print this picture so that you can see it every day. | - |
| *Notes.*  a. Instructions based on Albers et al. [54]  b. Instructions translated from Dutch. All participants first completed the desired future-self task and then the undesired future-self task. The order of the verbal and visual tasks were counterbalanced. | | | |
