# Supplementary material for: User Experiences With Digital Future-Self Interventions in the Contexts of Smoking and Physical Inactivity: Mixed Methods Multistudy Exploration
Source: JMIR Form Res. 2025 Jun 20;9:e63893. doi: 10.2196/63893 (PMC12228003; doi:10.2196/63893)
Supplement: Multimedia Appendix 2 [file formative_v9i1e63893_app2.docx]

# **Supplement 2 – Results of assumption testing and robustness analyses**

## Study 1

### Results of assumption testing

For anticipated difficulty, Shapiro-Wilk tests showed violation of normality but visual inspection of Q-Q plots did not. For anticipated completion time, both Shapiro-Wilk and visual inspection of Q-Q plots showed violation of normality. Despite some violations of normality, no transformations were applied due to the robustness of ANOVAs with samples larger than 30-40 [88]. Three and thirteen outliers, with a length of at least 1.5 from the edge of the boxplot, were identified for anticipated difficulty and anticipated completion time respectively. Analyses were conducted with and without for robustness. For anticipated difficulty, results including outliers were leading for conclusions, but for anticipated completion time, results excluding outliers were leading due to a clear violation of normality when including them.

### Results of robustness analyses

#### Anticipated completion time for future-self tasks

When including the 13 outliers, mean anticipated completion time was 14.78 minutes (*SD=*7.88). After Bonferroni corrections, analyses with these outliers did not reveal a significant 3-way interaction between behavior, polarity and modality, *F*(1,143)=1.21, *P=*.273, η_p_^2^=.008. They did reveal a significant two-way interaction between behavior and modality, *F*(1,143)=4.58, *P=*.034, η_p_^2^=.031, and simple main effects. Specifically, describing the smoking-related desired future-self verbally was anticipated to take more time than describing it visually (*F*(1,143)=26.33, *p* < .001, η_p_^2^=.156), describing the PA-related desired future-self verbally was anticipated to take more time than describing it visually (*F*(1,143)=22.11, *p* < .001, η_p_^2^=.145), describing the smoking-related undesired future-self verbally was anticipated to take more time than describing it visually (*F*(1,143)=11.42, *p* < .001, η_p_^2^=.074) and describing the PA-related undesired future-self verbally was anticipated to take more time than describing it visually (*F*(1,143)=23.23, *p* < .001, η_p_^2^=.140).

#### Anticipated difficulty of future-self tasks

The three-way interaction between behavior, polarity and modality was not significant when excluding the three outliers in the analyses, *F*(1,141)=3.63, *p*=.059, η_p_^2^=.025. In line with results including outliers, after Bonferroni corrections, there were no significant two-way interactions between behavior and polarity, *F*(1,141)=0.50, *p*=.825, η_p_^2^=.000, between behavior and modality, *F*(1,141)=1.95, *p*=.165, η_p_^2^=.014, or between polarity and modality, *F*(1,141)=2.27, *p*=.134, η_p_^2^=.016. Similarly, in analyses without outliers, there was a main effect of polarity, *F*(1,141)=38.78, *p*<.001, η_p_^2^=.217, with desired future-self tasks anticipated to be easier than undesired future-self tasks, ∆=0.98, 95% CI [0.66,1.23].

## Study 2

### Results of assumption testing

Shapiro-Wilk tests showed violations of normality for effort, but visual inspection of Q-Q plots did not. No transformations were applied as explained in Study 1. Five outliers with a length of at least 1.5 from the edge of the boxplot were identified, therefore analyses were conducted with and without for robustness. Analyses with outliers were leading for drawing conclusions.

### Results of robustness analyses

### *Effort deployed on future-self tasks* Aligned with results including the nine outliers, there was no significant three-ways interaction between behavior, polarity and modality, F(4,415)=8.11, p=.239, ηp2=.013. After Bonferroni corrections, there were also no two-way interactions between behavior and polarity, F(4,415)=3.27, p=.371, ηp2=.010, between behavior and modality, F(4,415)=7.48, p=.279, ηp2=.013, or between polarity and modality, F(4,415)=1.38, p=.240, ηp2=.013. There was no significant main effects of behavior, F(2,435)=0.19, p=.826, ηp2=.001, polarity, F(2,435)=0.95, p=.389, ηp2=.004, or modality, F(2,435)=0.85, p=.428, ηp2=.004.

## Study 3

### Results of assumption testing

Shapiro-Wilk tests showed violations of normality for experienced difficulty and emotional response, but visual inspection of Q-Q plots did not. For time spent, both Shapiro-Wilk tests and visual inspection of Q-Q plots showed violation of normality. No transformations were applied as explained in Study 1. Zero, five and five outliers, with a length of at least 1.5 from the edge of the boxplot, were identified for experienced difficulty, time spent and emotional response respectively. Analyses were conducted with and without for robustness. Analyses with outliers were leading in the conclusions regarding experienced difficulty and emotional response, but for time spent, results excluding outliers were leading due to a clear violation of normality when including them.

### Results of robustness analyses

### *Time spent on future-self tasks*

Contrary to analyses excluding the five outliers, analyses with outliers revealed no significant interaction effect of polarity and modality (F(1,86)=0.57, p=.452, ηp2=.007), and no main effect of polarity (F(1,86)=2.57, p=.113, ηp2=.029) or modality (F(1,86)=2.46, p=.121, =.028).

### *Emotional response to future-self tasks*

Akin to analyses with outliers, there was a significant main effect of polarity on emotional response to future-self tasks, *F*(4,78)=35.33, *p* < .001, η_p_^2^=.644. Specifically, the desired future-self task elicited significantly more happiness than the undesired future-self (FS) task, *F*(1,81)=139.68, *p* < .001, η_p_^2^=.633. The undesired FS task elicited significantly more anger (*F*(1,81)=74.07, *p*<.001, η_p_^2^=.478), sadness (*F*(1,81)=85.43, *p* < .001, η_p_^2^=.513), and anxiety (*F*(1,81)=33.390, *p*<.001, η_p_^2^=.292).
