# Supplementary material for: User Experiences With Digital Future-Self Interventions in the Contexts of Smoking and Physical Inactivity: Mixed Methods Multistudy Exploration
Source: JMIR Form Res. 2025 Jun 20;9:e63893. doi: 10.2196/63893 (PMC12228003; doi:10.2196/63893)
Supplement: Multimedia Appendix 3 [file formative_v9i1e63893_app3.docx]

# **Supplement 3 – Coding trees for qualitative analyses**

## Study 1 – Cognitive experiences with a PA- and smoking-related future-self intervention

Survey question: ‘Think of the preparatory activities that you have just seen. What makes an activity more difficult than others?’

1. ANTICIPATED TIME INVESTMENT
2. ANTICIPATED REQUIRED EFFORT
   1. Mental / cognitive
   2. Physical
3. TASK FORMAT
   1. Task interpreted as asking to take action
      1. Being physically active
   2. Multi-component task
   3. Task requires passive processing of information
4. CHARACTERISTICS RELATED TO PARTICIPANT
   1. Personal characteristics
      1. Being honest / candid with oneself
      2. Differences in which polarity is more difficult
      3. Motivation to do the task / change behavior
5. TASK CONTENT
   1. Visualizing / Thinking
      1. Visualizing in general
      2. About the future (future-self, future events, future behavior)
   2. Watching video’s
   3. Reading
   4. Searching for information
   5. Recording information
6. SUBSTANTIATION FOR ANTICIPATED DIFFICULTY/EASE OF FUTURE-SELF TASKS
   1. No substantiation
   2. Tasks requires cognitive effort rather than passive processing of information
   3. Attainability of future-selves
   4. Changing behavior / habits / identity
   5. Negative emotional response to task
   6. Familiarity with / Experience with task
   7. Visualization more difficult than taking concrete steps to change behavior

## Study 2 – Behavioral and cognitive experiences with a PA- and/or smoking-related future-self intervention

Survey question: ‘How did you approach, do, or experience your assigned activity?’

## BEHAVIORAL EXPERIENCES

1. TASK EXECUTION AND COMPLETION
   1. Conditions in which to complete the task
   2. Time of day
   3. Frequency
   4. Unable to complete task
   5. Personalized task to own needs / life
   6. Looked at generated materials after task completion
2. TASK OUTCOME
   1. Behavior change
   2. Seeking information about smoking/PA
   3. Formulated goal(s)
   4. Formulated action plan to reach goal(s)

## COGNITIVE EXPERIENCES

1. GENERAL TASK EXPERIENCE
   1. FS task (very) difficult
   2. FS task not difficult / easy
2. TASK EXECUTION AND COMPLETION
   1. Resistance / refusal to complete task
3. TASK OUTCOME
   1. Comparison / contrasting of future-selves
   2. Confrontation with fears / negative outcomes or aspects of behavior
   3. Found role model
   4. Thinking about consequences of current and changed behavior

## AFFECTIVE EXPERIENCES

1. TASK OUTCOME
   1. Emotional response to task

## PERSONAL CHARACTERISTICS REPORTED AS INFLUENCING TASK COMPLETION

- 1. Hindrance to task completion
     1. Personality trait (e.g., refusing to think about negative things)
     2. Does not perceive urgency to change behavior
  2. Facilitated task completion
     1. Familiarity with task

## Study 3 – Behavioral, cognitive and affective experiences with a smoking-related future-self intervention

Survey questions: ‘What was it like for you to perform the tasks about yourself in a future when you have [successfully quit/continued] smoking?’

## BEHAVIORAL EXPERIENCES

1. TASK COMPLETION
   1. Saved generated materials

COGNITIVE EXPERIENCES

1. EXPERIENCED DIFFICULTY
2. TASK OUTCOME
   1. DESIRED FUTURE-SELF
      1. Altered motivation to quit smoking
      2. Considering consequences of current and changed smoking behavior
      3. Awareness of reasons to quit
      4. Awareness of future (in general)
      5. Awareness of attitude towards smoking / Focus on positive aspects of smoking
      6. Awareness of negative aspects of smoking
      7. Goal / something to work towards
      8. Something to look forward to
      9. Self as quit smoker unreachable / unattainable
      10. Seeing the future in a more positive light
      11. Triggered reflection / thinking about
          1. Desired future-self
          2. Being a smoker
          3. Being a nonsmoker
          4. What is important in the future
      12. Cognitive dissonance
      13. Resistance
      14. Mental contrasting with past self
   2. UNDESIRED FUTURE-SELF
      1. Altered motivation to quit smoking
      2. Focus on positive aspects of smoking
      3. Confrontation with addiction / nicotine dependence
      4. Doesn't want to become this FS
      5. Felt pressured to be negative about oneself
      6. Summarizing images saved
      7. Increased awareness of consequences of smoking and current behavior
      8. Triggered reflection / thinking about
      9. Mental contrasting
      10. Envisioned self feels realistic
      11. No effect
   3. COMPARISON DESIRED/UNDESIRED FUTURE-SELF
      1. Same answer as other FS task
      2. Order (future-self as continued smoker first)
      3. As easy as envisioning self as quit smoker
      4. More difficult than envisioning self as quit smoker
      5. Easier than envisioning self as quit smoker
      6. Less important than self as successfully quit smoker

AFFECTIVE EXPERIENCES

1. EMOTIONAL RESPONSE TO DESIRED FUTURE-SELF TASK
2. EMOTIONAL RESPONSE TO UNDESIRED FUTURE-SELF TASK

## UNUSABLE
